# SLC22A5 (OCTN2) Carnitine Transporter—Indispensable for Cell Metabolism, a Jekyll and Hyde of Human Cancer

**DOI:** 10.3390/molecules25010014

**Published:** 2019-12-19

**Authors:** Barbara Juraszek, Katarzyna A. Nałęcz

**Affiliations:** Laboratory of Transport through Biomembranes, Nencki Institute of Experimental Biology of Polish Academy of Sciences, 3 Pasteur Street, 02-093 Warsaw, Poland; b.juraszek@nencki.edu.pl

**Keywords:** carnitine, SLC22A5/OCTN2, cancer

## Abstract

Oxidation of fatty acids uses l-carnitine to transport acyl moieties to mitochondria in a so-called carnitine shuttle. The process of β-oxidation also takes place in cancer cells. The majority of carnitine comes from the diet and is transported to the cell by ubiquitously expressed organic cation transporter novel family member 2 (OCTN2)/solute carrier family 22 member 5 (SLC22A5). The expression of *SLC22A5* is regulated by transcription factors peroxisome proliferator-activated receptors (PPARs) and estrogen receptor. Transporter delivery to the cell surface, as well as transport activity are controlled by OCTN2 interaction with other proteins, such as PDZ-domain containing proteins, protein phosphatase PP2A, caveolin-1, protein kinase C. *SLC22A5* expression is altered in many types of cancer, giving an advantage to some of them by supplying carnitine for β-oxidation, thus providing an alternative to glucose source of energy for growth and proliferation. On the other hand, SLC22A5 can also transport several chemotherapeutics used in clinics, leading to cancer cell death.

## 1. Introduction

Fatty acids fulfill various roles in the cell: they are components of membrane lipids, they regulate proteins in lipid posttranslational modifications, they control signal transduction pathways and, last but not least, they constitute an energy source in oxidation.

In healthy humans, oxidation of fatty acids takes place mainly in liver, but it is also very active in energy-demanding tissues, e.g., skeletal muscle and heart. This is why disorders of fatty acid oxidation result in symptoms like myopathy and progressive cardiomyopathy. Other symptoms have also been reported, such as neuropathy, hypoglycemic hypoketotic encephalopathy or Reye-like syndrome, seizures and mental retardation [1], which points to an involvement of β-oxidation in the physiological activity of the brain.

**Figure 1:**
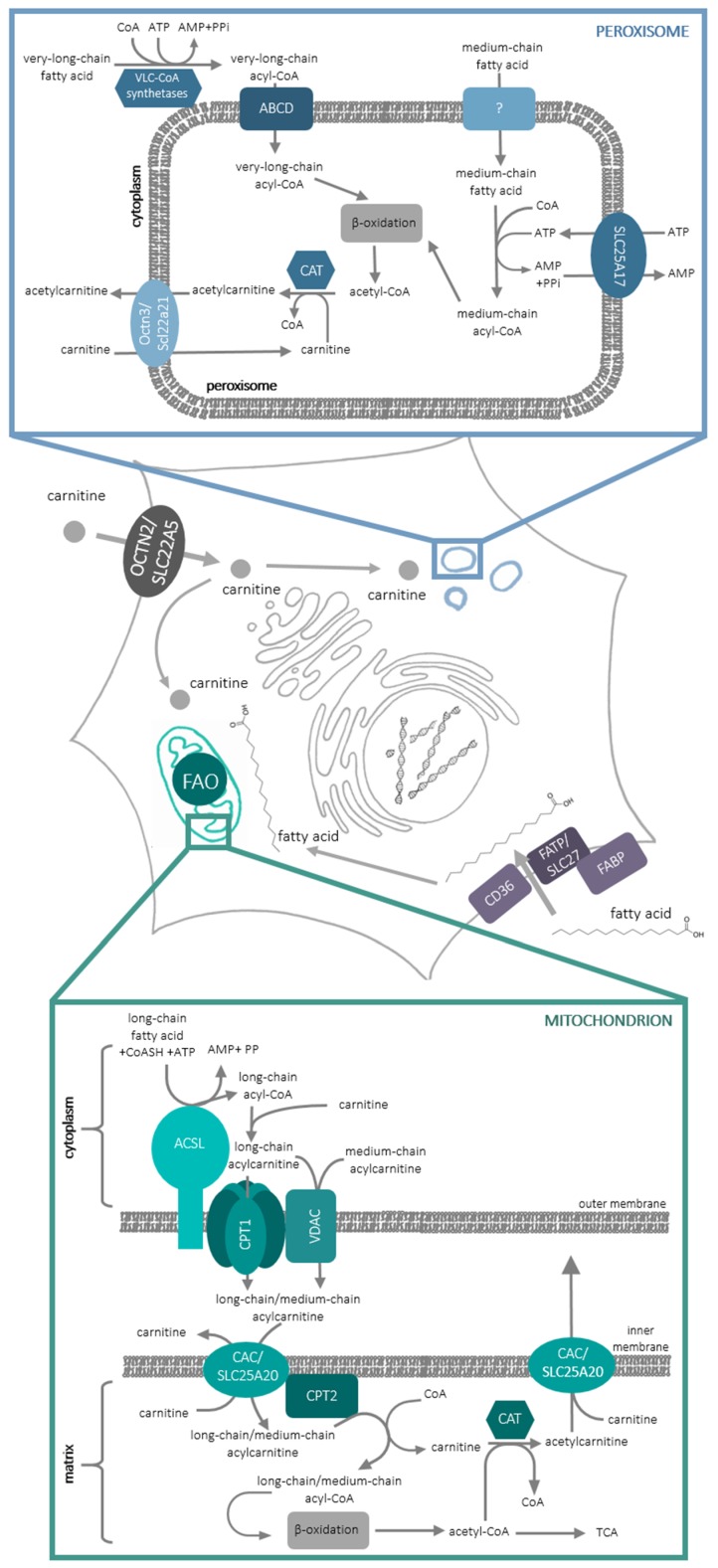
Fatty acid oxidation processes involving carnitine in mammalian cell. For a detailed description and abbreviations see the text. FAO, fatty acid oxidation.

At the molecular level, there exist two cellular compartments in which fatty acid oxidation occurs: peroxisomes and mitochondria. In peroxisomes, very-long-, branched-chain- and medium-chain fatty acids are oxidized (Figure 1) in the process of α- and β-oxidation (for review, see [2]). Long-chain fatty acids are oxidized inside mitochondria in the process of β-oxidation. In order to enter the oxidative pathway, fatty acids have to be activated to acylCoAs. Very-long chain fatty acids are activated on the cytoplasmic side of peroxisomes and are transported to these organelles by one of the transporters belonging to the ATP-binding cassette (ABC) subfamily D [3]. The mechanism of medium-chain fatty acids’ entry into peroxisomes still remains obscure; they are activated inside these organelles at the expense of ATP, transported to peroxisomes by solute carrier (SLC)-SLC25A17 (Ant1p/PMP3) transporter [4] in an exchange with AMP formed in the process of fatty acid activation. In the mitochondria, CoA derivatives of long-chain fatty acids are formed by acyl-CoA syntethase (ACLS), an enzyme containing one transmembrane domain and localized in the outer mitochondrial membrane. Since the CoA derivatives of fatty acids do not penetrate lipid bilayer, fatty acyl moiety is delivered to mitochondrial matrix thanks to the so-called carnitine shuttle (Figure 1).

l-carnitine, (*3R*)-3-Hydroxy-4-(trimethylazaniumyl)butanoate, contains 3 functional groups: trimethylamine-, carboxyl- and a hydroxyl group, which is capable of forming an ester bond with acidic compounds. The acyl moiety of long-chain acyl CoA is transferred to this hydroxyl group of l-carnitine by carnitine palmitoyltransferase 1 (CPT1) protein, which is also situated in the outer mitochondrial membrane. CPT1 can be inhibited by malonyl-CoA, first intermediate in fatty acid synthesis, thus making this enzyme a control switch between fatty acid catabolism and synthesis. CPT1 contains 2 transmembrane domains, with its N- and C-terminus and both the malonyl-CoA binding site and the active site, on the cytoplasmic side of the enzyme [5]. Such a location of the active site requires a mechanism responsible for acylcarnitine transport to the mitochondrial matrix. It is possible that acylcarnitines may cross the outer mitochondrial membrane through the mitochondrial Voltage-dependent anion channel (VDAC) [6]. Since CPT1 forms dimers, which can further oligomerize into hexamers [7], it was also proposed that acylcarnitines can cross the outer mitochondrial membrane through CPT1 hexamer. It is noteworthy that CPT1 was shown to interact with both ACLS and VDAC [8]. Acyl-carnitines are further transported through the inner mitochondrial membrane by carnitine-acylcarnitine carrier (CAC/SLC25A20) in an exchange reaction with free l-carnitine leaving the mitochondrial matrix [9,10]. CAC interacts with carnitine palmitoyltransferase 2 (CPT2), which is located on the inner side of the inner mitochondrial membrane and releases free l-carnitine and acyl-CoA, which can enter the β-oxidation pathway (Figure 1). It needs to be added that carnitine acyl transferases are also present in the peroxisomes [11] and, since the oxidation of fatty acids in these organelles is not complete, it was suggested that shortened fatty acids in the form of acylcarnitines can be exported from peroxisomes for further oxidation in mitochondria [11]. Slc22a21 (Octn3) was argued to catalyze this reaction [12].

## 2. Carnitine Transporters

Since carnitine is such an indispensable compound, not only for the translocation of fatty acids through the membranes but also for the regulation of acylCoA/CoA ratio, cells need a continued supply of carnitine. Carnitine biosynthesis begins with the methylation of lisyl residues in proteins and is followed by protein lysosomal degradation and further synthesis from trimethyllysine, which engages several enzymes (for review see [13]). Interestingly, the activity of the last enzyme in this pathway-γ-butyrobetaine dioxygenase- was detected exclusively in rat and mouse liver as well as in rat testis, while it was not detectable in the brain [14]. In humans, the endogenous synthesis is, however, insufficient and approximately 75% of carnitine is sourced from food, mainly from red meat [15]. Being a water-soluble zwitterion, carnitine has to use transporting proteins to cross the biological membranes. In accordance with the current classification of solute carriers (SLCs), the carnitine transporters belong to three different families: SLC22, SLC25 and SLC6 (Table 1).

There are two carnitine transporters functioning in the intracellular membranes: SLC25A20 and Slc22a21. The first one is a carnitine carrier of the inner mitochondrial membrane [9,25] responsible for carnitine/acylcarnitine exchange (Figure 1). Its structure, consisting of six transmembrane helices, is typical of other mitochondrial carriers [10]. The second one, as mentioned in the Introduction, was detected in peroxisomes [12,18]. Although its gene (*Slc22a21/Slc22a9*) was cloned from mouse [17], the protein was also detected in human fibroblasts [18]. It is argued that the human gene is located at 5q31 between *SLC22A4* and *SLC22A5* and is associated with Crohn disease [26]. Octn3 was also proved to be present in the rat brain, in the peroxisomes of astrocytes [12]; moreover, its expression was up-regulated by treatment with peroxisome proliferators-activator receptor (PPAR) agonist [12].

There are three carnitine transporters that are situated in the plasma membrane and as such can supply carnitine to the cells: SLC22A16, SLC6A14 and SLC22A5. However, they all differ in their affinities for carnitine and their expression in the human body.

Carnitine transporter 2 (CT2/FLIPT2) coded by human *SLC22A16* gene was identified [20] and it transports carnitine by facilitated diffusion. Although CT2 transports carnitine with a high affinity (*K*_m_ = 20.3 μM), its expression is limited since it is mainly expressed in testis and epidydimis [20].

SLC6A14 is also capable of transporting carnitine, though with a very low affinity (*K*_m_ = 830 μM) [21]. This is an amino acid transporter ATB^0,+^ specific for neutral (index “0”) and basic (index “+”) amino acids and transports its substrate with two Na and one Cl ions [27]. Considering much lower *K*_m_ values for amino acids [27], the involvement of SLC6A14 in carnitine uptake seems less probable, although mice with inactive Slc22a5/Octn2 accumulated carnitine in the brain [28], which might arise from the presence of Slc6a14 in the blood-brain barrier [29,30].

Apart from transporting organic cations, SLC22A5/OCTN2-organic cation transporter novel family member 2 is characterized by a high affinity for carnitine (*K*_m_ = 4.34 µM) [22]. Its activity is inhibited by short- and long-chain acylcarnitines [22,31]; therefore, it was also named CT1 (carnitine transporter 1). The loss of OCTN2 function causes systemic carnitine deficiency [32]. The human gene coding SLC22A5 was cloned in 1998 [22,23] and the protein was named OCTN2 because of its similarity (75.8%) to OCTN1/SLC22A4. Overexpressed OCTN1 was shown to transport carnitine in a Na^+^-dependent manner [17,33]; however, its activity was much lower in comparison with OCTN2 and OCTN3 [17]. Subsequently, OCTN1 was shown to be primarily an ergothioneine transporter [34].

**Figure 2:**
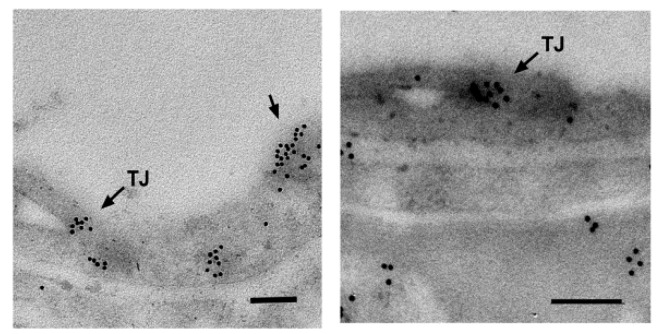
Presence of Octn2 in the blood-brain barrier. Rat brain slices were obtained, fixed and embedded in Epon after dehydration, as presented in [30]. They were subsequently treated with anti-OCTN2 antibody and the secondary antibody coupled to 10 nm gold particles, as presented in [12]. The areas with selected capillaries are shown. The TJ, tight junction; single arrow, Octn2 in the apical membrane. Octn2 is also detected in astrocytic endfeet (right panel). The bar size: 200 nm.

What is more, SLC22A5/OCTN2 is ubiquitously expressed [22,23,31], which renders it the principal plasma membrane carnitine transporter. In addition, it is expressed in the brain; a transcriptome analysis using Affymetrix GeneChip Arrays demonstrated *Slc22a5* expression in various brain cell types: astrocytes, neurons and oligodendrocytes [35]. The level of OCTN2 RNA expression was proved to increase during development in various brain regions [36]. It ought to be added that SLC22A5/OCTN2 was also detected in the brain at the protein level; it is located in the capillary endothelial cells forming the blood-brain barrier (Figure 2) [29], in astrocytes [12] and in neurons [24].

Recently, during the differentiation of human monocytes to macrophages, a possibility of carnitine transport by another amino acid transporter—SLC38A/SNAT2 was proposed, due to a correlation between an increased expression of this transporter and carnitine transport [37]. However, there is no conclusive evidence that would follow from an over-expressed transporter.

## 3. SLC22A5 Regulation

**Figure 3:**
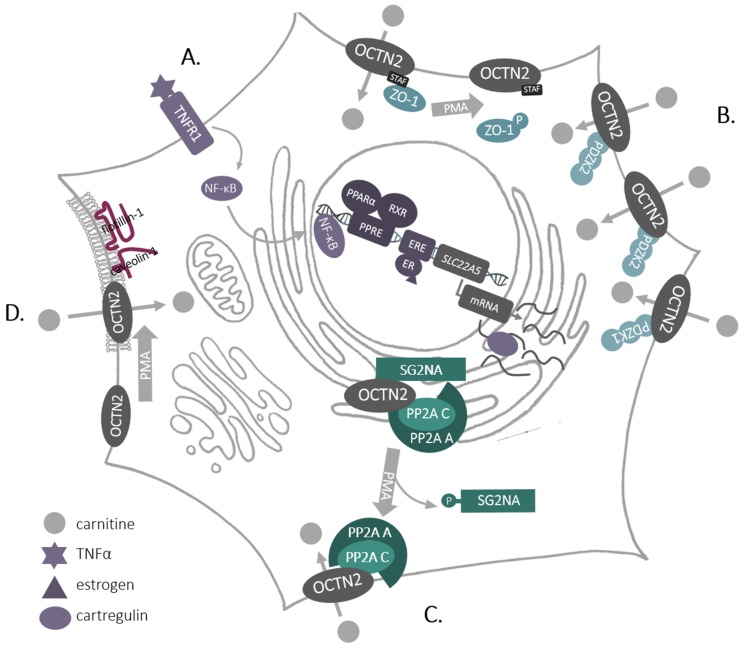
Regulation of SLC22A5/OCTN2 in the mammalian cell. **A**. Transcription of *SLC22A5*; **B**. Interaction of SLC22A5/OCTN2 with PDZ proteins; **C**. Trafficking to the plasma membrane-interaction with phosphatase PP2A; **D**. Lateral movement in the plasma membrane to rafts. For a detailed description and abbreviations see the text.

SLC22A5/OCTN2 is the only high-affinity carnitine transporter that is located in the plasma membrane and is ubiquitously expressed. Therefore, it is strictly regulated—from transcription and translation—through posttranslational modifications and interactions with other proteins at various steps of protein trafficking to the plasma membrane (Figure 3).

Expression of *SLC22A5* is regulated by estrogens, and an estrogen receptor responsive element was found in its first intron [38]—an interesting observation bearing in mind the role of estrogen signaling in breast cancer. *SLC22A5* transcription is also significantly enhanced by agonists of nuclear receptors—peroxisome proliferator-activated receptors (PPARs) [39,40,41,42], (Figure 3A) and the PPARα responsive element was also located to the first intron of *SLC22A5* [43]. This was confirmed for other species, including humans [44]. Furthermore, *SLC22A5* expression was stimulated by pro-inflammatory cytokines, including tumor necrosis factor α(TNFα), and an involvement of a nuclear factor KB (NF-KB) was suggested [45]. What is more, during the differentiation of human monocytes to macrophages by granulocyte-macrophage colony stimulating factor, increased expression of *SLC22A5* correlated with increased phosphorylation of mTOR kinase and activation of the transcription factor STAT3 [37].

The transcript of rat *Slc22a5* was shown to be stabilized in endoplasmic reticulum (ER) through co-injection to *Xenopus* oocytes of cRNA coding cartregulin, a protein highly homologous to the first 146 amino acids of OCTN2 [46]. This co-expression also resulted in an elevated level of OCTN2 and an enhanced transport activity [46].

Being a plasma membrane protein, SLC22A5 is inserted co-translationally into the ER membrane, it is glycosylated in ER and Golgi at the first extracellular loop [47] and delivered in vesicular transport to the cell surface. This process was shown to be regulated by activation of protein kinase C (PKC) [48], although the transporter itself is not phosphorylated by this kinase [48,49]. At SLC22A5 C-terminus the last 4 amino acids STAF were proved to be motif binding proteins with so-called PDZ domains (named after proteins: postsynaptic density 95/disc large/zonula occludens-1). The pull-down experiments and immunochemistry analyses in the kidney brush-border membranes displayed co-localization of OCTN2 with PDZK1 (Na^+^/H^+^ exchange regulatory cofactor NHE-RF3), a phenomenon not observed after truncation of the last 4 amino acids [50]. Co-transfection with genes coding the both proteins led to a substantially enhanced transporting activity, without any change in OCTN2 surface presence. The same group revealed that co-expression of OCTN2 with PDZK2 (intestinal and kidney-enriched PDZ protein—IKEEPP, Na^+^/H^+^ exchange regulatory cofactor NHE-RF4) resulted in the augmented transport activity and in the surface presence of the transporter [51]. A mass spectrometry analysis of SLC22A5 interactome in rat astrocytes allowed to identify, two PDZ proteins out of 156 proteins: zonula occludens-1 (ZO-1) and AHNAK [52]. In addition, experiments with overexpressed SLC22A5 exhibited co-localization with ZO-1 and no direct interaction with AHNAK [53], which can suggest that AHNAK may be a part of a bigger complex, especially because PDZ proteins are known to interact with one another (for review, see [54,55]). What is noteworthy, phosphorylation of ZO-1 by PKC as well as depletion of the STAF motif resulted in decreased carnitine transport [53], which led to a conclusion that non-phosphorylated ZO-1 maintains the transporter in its active state. The aforementioned observations point to the fact that the activity of SLC22A5 can be controlled by PDZ proteins (Figure 3B), similarly to the control of channel proteins [56].

However, phosphorylation of ZO-1 did not affect the amount of SLC22A5 in the plasma membrane [53] and the trafficking of the transporter was shown to be controlled by PKC through protein phosphatase PP2A (Figure 3C), detected in SLC22A5 intaractome [52]. PP2A, as a complex containing structural subunit A, catalytic subunit C and the regulatory subunit SG2NA, co-localizes with OCTN2 in the ER, while activation of PKC results in phosphorylation of the regulatory subunit SG2NA, leading to the transfer of the transporter with A and C PP2A subunits to the cell surface. This enables a carnitine uptake by the cell [52]. What is more, activation of PKC fosters a lateral movement of SLC22A5 in the plasma membrane, increasing the amount of the transporter in cholesterol-rich domains (rafts) and augmented interaction with caveolin-1 and flotillin-1 [48] (Figure 3D). Experiments with the deletion mutants proved a direct interaction between caveolin-1 and amino acids 14–22 and 447–454 of rat Octn2 sequence [48]. All these observations demonstrate that SLC22A5 can be regulated by other proteins at various steps: exporting from the ER, trafficking to the plasma membrane and locating in microdomains, what affects transporter activity. Since activators of PKC, e.g., phorbol esters, are tumor promoters [57], while PP2A is considered to be a tumor suppressor [58], such comprehensive regulation of SLC22A5 may have implications in pathological states, in particular in cancer.

## 4. SLC22A5 Pharmacological Implications

Apart from carnitine, SLC22A5 can also transport organic cations, in a sodium-independent way [22] and pharmacologically active compounds [22,59]. Said compounds include e.g., verapamil, an anti-arhytmia agent [60], which is not surprising given the role of SLC22A5 and fatty acid oxidation in heart function. Interestingly, however, there are also clinically used anti-psychotic drugs (amisulpride [61]) and anti-cancer drugs such as etoposide [62], oxaliplatin [63] and imatinib [64] that are taken up by cells via SLC22A5 (Table 2). Even more drugs were proven to inhibit carnitine uptake by cells, acting as an SLC22A5 inhibitors (Table 3), including several anticancer drugs as well. Carnitine and its derivatives are believed to have neuroprotective properties; that is why there are several clinical trials underway studying whether carnitine can reduce neurotoxicity in cancer patients undergoing chemotherapy (https://clinicaltrials.gov/). Some researches established that even though there was no difference in overall peripheral neuropathy incidence between patients treated with combination of sagopilone and acetyl-l-carnitine and patients treated with sagopilone and placebo, the severity of this neuropathy was significantly lower in patients who were given Acetyl-l-carnitine [65]. On the other hand, it was revealed that Acetyl-l-carnitine in the long term could even increase taxane-induced neuropathy in women undergoing adjuvant breast cancer therapy [66]. This points to a need for a deeper understanding of the role played by carnitine, SLC22A5 and fatty acid oxidation in both cancer and in the brain—fields that up until recently have been under-researched.

## 5. SLC22A5 and Fatty Acid Oxidation in the Brain

For many years, glucose has been considered the main energetic substrate of the adult brain [78,79], although lactate delivered by astrocytes was postulated to be an alternative energy source for activated neurons [80]. In 2003, Ebert et al. [81] demonstrated in nuclear magnetic resonance (NMR) studies that the brain of adult rat can oxidize ^13^C-octanoate, a process detected in astrocytes, and that this process accounts for 20% of the brain oxidative energy. Nonetheless, the utilization of fatty acids by the brain in energy delivery has remained controversial [82]. Oleate, a long-chain fatty acid, was suggested to cross the blood-brain barrier as well [83]. Moreover, the brain’s ability to accumulate fatty acids was further evidenced through the detection of fatty acid transporters in the brain microvessel endothelial cells: fatty acid transport proteins (FATPs) 1 and 4, fatty acid binding protein 5 and fatty acid translocase/CD36 [84]. In 2017, Jernberg et al. [36] confirmed that four enzymes crucial for fatty acid oxidation-CPT1, CPT2 and two acyl-CoA dehydrogenases specific for either long-chain or medium-chain acyl-CoAs, as well as SLC22A5/OCTN2– are all expressed in various areas of the rat brain, with the expression of the last three even increasing throughout the development. The authors further demonstrated the capability of [1-^14^C]oleate oxidation in dissected cortex and hippocampus, as well as proved sensitivity of this process to etomoxir, an inhibitor of CPT1. Immunofluorescence experiments with the cell-type specific markers showed that CPT1a is found exclusively in astrocytes (GFAP-positive) and neural progenitor cells (Nestin-positive), although it was not detected in neurons, oligodendrocytes or microglia [36]. In addition, OCTN2 appears to be essential for the functioning of the brain; a deletion in *SLC22A5* was reported in the attention deficit/hyperactivity disorder [85], and the deficiency of carnitine biosynthesis was revealed to be a risk factor in autism [86] with improvement after carnitine supplementation [87].

## 6. SLC22A5 and Fatty Acid Oxidation in Cancer

Interestingly, the fatty acid oxidation enzymes (including CPT1a) were also detected in glioma cells and the process of fatty acid oxidation contributed significantly to their aerobic respiration, while treatment with etomoxir decreased cell proliferation and prolonged survival in the mouse model of this disease [88]. Moreover, OCTN2 expression was increased in primary glioblastoma samples from patients, and even more so in samples from patients with recurrent glioblastoma, when compared to the healthy brain [89].

Metabolic reprogramming is now widely recognized as one of the so-called hallmarks of cancer, onset of traits, by which cells undergoing malignant transformation are characterized [90]. The uptake of glucose by cancer cells is high due to up-regulation of facilitative glucose transporter GLUT1 [91,92]. In cancer cells glucose is mainly metabolized in glycolysis and is preferentially used for synthesis of ribose, serine or protein glycosylation, which led to the hypothesis of malfunctioning of mitochondria (the so-called Warburg effect). Lactate, as an end product of glycolysis causes acidification of the microenvironment. Although researchers paid little attention to the process of fatty acid oxidation in the context of cancer cells, more and more papers are published which demonstrate that fatty acids that are taken up by cancer cells or come from hydrolyzed triglycerides can deliver ATP (for review, see [93]). The growth of cancer cells depends on the availability of NADPH, and the process of fatty acid oxidation delivers this co-enzyme for many anabolic processes [93]. Treatment of human glioblastoma cells with etomoxir, a CPT1 inhibitor, results in a decreased level of NADPH, depletion of ATP and an increased level of reactive oxygen species [94]. Furthermore, inhibition of fatty acid oxidation results in an increased level of cytotoxic lipids, such as proapoptotic ceramides containing palmitic and stearic acid, which in turn reduces mTOR signaling and rises the level of active caspase-3 [95]. What is more, cancer cells that oxidize fatty acids are resistant to radiation [96]. One needs to note that changes in cancer cells’ metabolic pathways require transcriptional control. It was shown that in prostate cancer, among 10 co-regulators of a metabolic switch, the peroxisome proliferator-activated receptor gamma co-activator 1 alpha (PGC1α)—regulating the activity of estrogen-related receptor alpha (ERRα)—was identified, as well as that downregulation of PGC1α promoted metastasis and disease progression [97]. PGC1α was shown to induce transcription of several genes coding proteins involved in metabolic pathways, including those engaged in fatty acid catabolism [97]. Moreover, the activity of PGC1α-ERRα negatively correlated with levels of oncogenic transcription factor MYC [98]. However, it has to be added that the effect of PGC1α differs across various tumors (for review, see [99]). A metabolomics analysis of triple-negative breast cancer cells with an elevated level of MYC demonstrated a four-fold increase in production of palmitoylcarnitine when compared to control mammary gland. The upregulation of many activators of fatty acid oxidation and the downregulation of many activators of fatty acid synthesis were also detected [100]. In addition, a decrease in *ACACB* coding acetyl-CoA carboxylase beta synthesising malonyl-CoA, a CPT1 inhibitor, was correlated with a worse prognosis in patients, while the proliferation rate of etomoxir-treated triple-negative cells was reduced [100].

All these reports give rise to a conclusion that cancer cells are dependent on lipid oxidation for their survival and proliferation; hence CPT1 is now recognized as a promising and potential target in anticancer therapy. However, available CPT1 inhibitors—etomoxir, oxfenicine and perhexeline—all cause side effects like neuropathy or hepatitis. Even though perhexeline is now an approved anti-angina agent in Australia, New Zealand and Asia, neither of the other two (etomoxir and oxfenicine) were successfully tried in human clinical trials, with etomoxir being even rejected from clinical trials due to the severity of hepatotoxic side effects [101]. Bearing in mind that CPT1 requires carnitine for its function, it is worth taking a closer look at SLC22A5, a transporter that delivers carnitine into the cells, in the context of cancer.

OCTN2 expression is altered in many types of cancer. It was shown to be down-regulated in virus and nonvirus-mediated epithelial cancers, and methylation of its promoter was proposed as a regulatory mechanism [102]. On the other hand, the expression of *SLC22A5* was shown to be higher in high grade serous epithelial ovarian cancer [103]. An analysis with the use of CANCERTOOL [104] revealed that SLC22A5 expression was significantly lower in colorectal cancer than in normal tissue, which was also the case in the breast cancer. In lung adenocarcinoma, on the other hand, *SLC22A5* expression was up-regulated when compared to the healthy lung.

A mutational analysis of metastatic breast cancer resulted in the identification of several driver mutations affecting transcription factors which regulate metastatic genes. *SLC22A5* was among the mutated genes enhancing cancer cell migration [105]. This may be due to the presence of an intronic estrogen-response element (ERE) [38]. An analysis with the use of Human Protein Atlas (https://www.proteinatlas.org/humanproteome/pathology), a visualization tool for among other things The Cancer Genome Atlas (TCGA) datasets, demonstrates *SLC22A5* expression in several cancers, including glioma, breast cancer and endometrial cancer, with the highest level of expression in renal cancer. The prognosis of overall survival correlated with *SLC22A5* expression was either favorable or unfavorable, depending on the type of cancer (Table 4), with the biggest impact on patients’ prognosis in pancreatic, renal and endometrial cancer.

**Figure 4:**
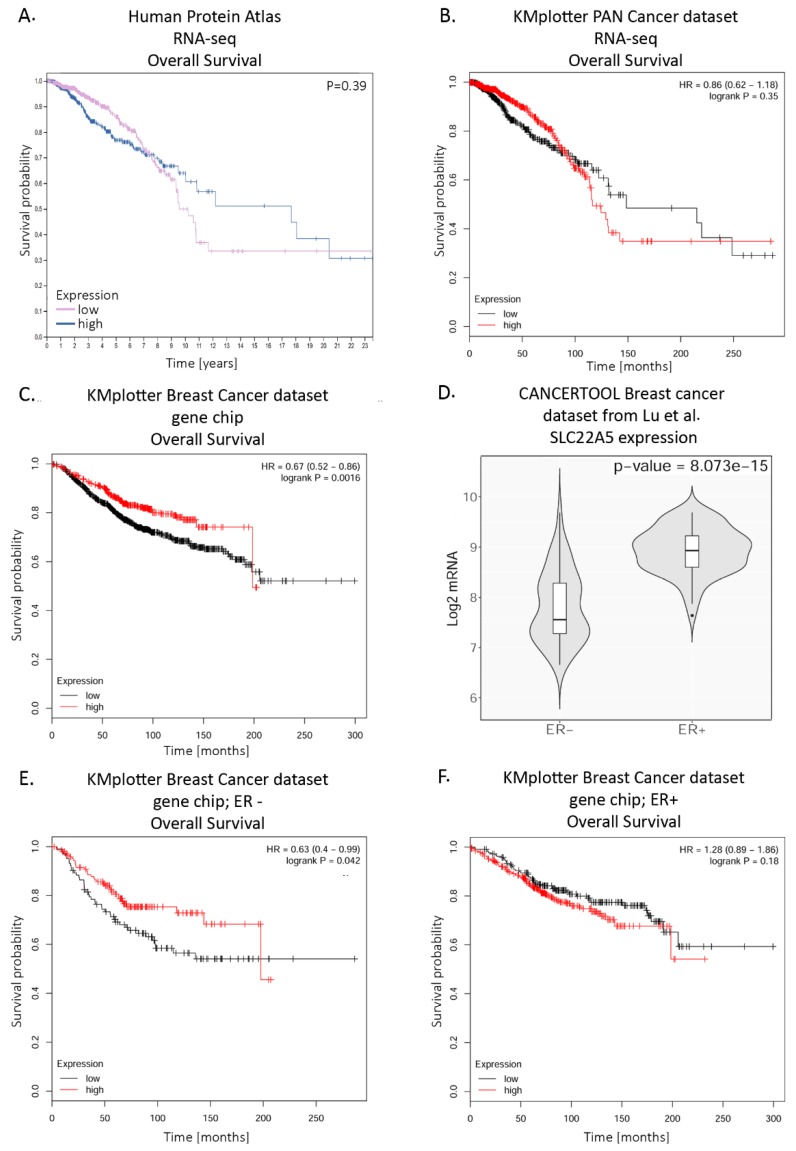
Probabulity of overall survival (OS) in breast cancer patients expressing high or low SLC22A5 levels. **A**. OS from Human protein Atlas with auto-selected best cutoff; mRNA data collected with RNA seq; **B**. OS assessed with the use of KMplotter and its PAN Cancer RNA seq dataset, with auto-selected best cutoff; **C**. OS assessed with the use of KMplotter and its Breast Cancer Affymetrix gene chip dataset, with auto-selected best cutoff; **D**. SLC22A5 expression in breast cancer ER− and ER+ samples from CANCERTOOL and dataset from [105]; **E**. OS assessed with the use of KMplotter and its Breast Cancer Affymetrix gene chip dataset restricted only to ER− patients, with auto-selected best cutoff; **F**. OS assessed with the use of KMplotter and its Breast Cancer Affymetrix gene chip dataset restricted only to ER+ patients, with auto-selected best cutoff. Graphic illustrations taken from proteinatlas.org, web.bioinformatics.cicbiogune.es/CANCERTOOL/and Kmplot.com.

Interestingly, a prognosis for breast cancer is better during the first 7 years for patients with a high *SLC22A5* expression level; after this period, it is the other way round and a prognosis is much better in case of patients with a low expression level (Figure 4A). Another analysis of probability of overall survival in breast cancer patients with the use of KMplotter tool (https://kmplot.com/analysis/) revealed varying outcomes depending on which dataset and assay for gene expression data acquisition were employed. A dataset from PAN cancer expression data showed a similar prognosis to the one in Human Proteome Atlas (Figure 4B). However, the dataset with breast cancer patients’ gene expression data collected with Affymetrix gene chip showed a prognosis for overall survival was significantly better for patients with high expression level of *SLC22A5* (Figure 4C). Knowing from Wang et al. [38] that *SLC22A5* expression is much higher in ER positive breast cancer than in ER negative one, also confirmed by Lu et al. [106], we further restricted this analysis to differentiate survival prognoses across ER+ and ER− patients (Figure 4E, 4F). Interestingly, there was no significant difference in survival of the ER+ patients with high or low expression of SLC22A5. In the ER− patients, however, those with higher expression of SLC22A5 had a much better prognosis than those with low SLC22A5 expression and survival curve for the former was similar to those of ER+. This might mean that patients with breast cancer that have reached a certain threshold of *SLC22A5* expression have a better prognosis regardless of ER status.

What is more, OCTN2 is ubiquitously expressed, including intestinal tract, hence it may play an important role in oral delivery of drugs. Due to a high affinity of l-carnitine for OCTN2, carnitine conjugates with several pharmacologically active compounds were tested in model systems to target some human diseases (for a review, see [107]). OCTN2 is also present in the blood brain barrier [29] and l-carnitine-conjugated poly(lactic-co-glycolytic acid) nanoparticles were shown to undergo transcytosis through this barrier and to be taken-up by glioma cells through OCTN2, which could improve the anti-glioma treatment [108].

In conclusion, l-carnitine is necessary for oxidation of fatty acids and is transported to the cells mainly by SLC22A5/OCTN2 protein, which is capable of transporting several drugs. This beneficial role of OCTN2 delivers energy to the cell. Fatty acid oxidation occurs not only in muscles and liver, but also in energy demanding cells like astrocytes and cancer cells, which can give an advantage to the latter by providing an alternative to the glucose source of energy for growth and proliferation. On the other hand, OCTN2 can transport several anti-cancer drugs leading to cell death. Moreover, OCTN2’s very high affinity towards carnitine can be utilized for better drug delivery with the use of drug-carnitine conjugates or carnitine-coated particles.

## Figures and Tables

**Table 1:** Mammalian carnitine transporters.

Name	Aliases	Type of Transport	Other Ions	*K*_m_ (µM)	Cell Localization	Expression	Ref.
**SLC25A20**	CAC, CACT	E, U	-	10200# (in)	inner mitochondrial membrane	ubiquitous	[16]
				480# (out)			
Slc22a21 *	Octn3	U	-	2.99	peroxisomal membrane	testis, brain, fibroblasts	[12,17,18,19]
**SLC22A16**	CT2/FLIPT2	F	-	20.3	plasma membrane	testis, epididymis, endometrium	[20]
**SLC6A14**	ATB^0,+^	U	2 Na^+^, 1 Cl^−^	803	plasma membrane	lung, trachea and salivary gland	[21]
**SLC22A5**	OCTN2, CT1	U	Na^+^	4.3	plasma membrane	ubiquitous; kidney, skeletal muscle, heart, placenta, brain	[12,19,22,23,24]

* mouse protein; ^#^ measurements after reconstitution in liposomes; E—exchange, U—uniport; F—facilitated transport.

**Table 2:** Transport of drugs by OCTN2/SLC22A5.

Drug	Drug Target and Use		Conc.	Net Uptake	Time of Uptake	Type of Assay	Other Info	Ref.
amisulpride	selective dopamine antagonist; antipsychotic drug, treatment of psychoses, schizophrenia and persistent depressive disorder	hOCTN2	5 µM	≈13 ± 1.5 pmol/min/mg protein	2 min	HPLC	Km 185.3 ± 68 µM	[61]
[^14^C]colistin	surface active agent which penetrates into and disrupts the bacterial cell membrane; antibiotic effective against most Gram-negative bacilli, particularly *Pseudomonas aeruginosa*	hOCTN2	1 µM	2.4 pmol/mg protein	10 min	radioactive		[67]
[^3^H]etoposide	DNA topoisomerase II inhibitor; treatment of testicular cancer, small cell lung cancer, acute myeloid leukaemia, lymphoma, ovarian cancer	hOCTN2	ND	2.85 µL/mg/5min	5 min	radioactive	independent of Na+; *K*m 150 ± 34.1 µM	[62]
mildronate	inhibitor of gamma-butyrobetaine dioxygenase, inhibits carnitine biosynthesis; anti-ischemia medication	hOCTN2	10 µM	≈250 µL/min mg protein	1 min	LC/MS/MS		[68]
[^14^C]oxaliplatin	cross-links DNA, thus inhibiting DNA synthesis and transcription; treatment of colon and rectum advanced carcinoma	hOCTN2	1 µM	≈1.5 µL/mg protein	1 h	radioactive		[63]
[^14^C]oxaliplatin		rOctn2	1 µM	≈3.5 µL/mg protein	1 h	radioactive		
[^3^H]verapamil	calcium channel blocker; class IV anti-arrhythmia agent used to treat hypertension, angina (chest pain), and certain heart rhythm disorders	mOctn2	12 nM	275 µL/mg protein	5 min	radioactive		[60]
[^3^H]pyrilamine	H1 receptor; antihistamine		50 nM	118 µL/mg protein	5 min	radioactive		
[^3^H]imatinib	Bcr-Abl tyrosine-kinase inhibitor; cancer treatment (chronic myelogenous leukemia (CML), acute lymphoblastic leukemia (ALL), gastrointestinal stromal tumors (GISTs) and other)	mOctn2	0.2 µM	140% of control	ND	radioactive		[64]

ND—no data.

**Table 3:** Effect of drugs on inhibiting carnitine transport.

Drug	Drug Target and Use	Concentration µM			Relative Uptake % of Control			Experimental System	Ref.
		hOCTN2	rOctn2	mOctn2	hOCTN2	rOctn2	mOctn2		
cephaloridine	disrupts the synthesis of the peptidoglycan layer of bacterial cell walls; experimental drug, withdrawn from clinical trials	2500			10			JAR; E	[69]
		2500	2500		13	25		HEK293; O	[69]
cefepime	disrupts the synthesis of the peptidoglycan layer of bacterial cell walls; active against Gram+ and Gram− bacteria, for the treatment of pneumonia, urinary tract infections, skin infections	2500			19			JAR; E	[69]
		2500	2500		27	28		HEK293; O	[69]
cefluprenam	disrupts the synthesis of the peptidoglycan layer of bacterial cell walls;	2500			58			JAR; E	[69]
		2500	2500		74	76		HEK293; O	[69]
nelfinavir mesylate hydrate	HIV-1 protease inhibitor; antiviral drug used for treatment of HIV	11.87 *	3.138 *		50	50		CHO; O	[70]
ipratropium bromide	blocks muscarinic acetylcholine receptors; anticholinergic agent for treatment of chronic obstructive pulmonary disease (COPD) and asthma	100			71.3 ± 2.3			MDCKII; O	[71]
		100/1000			38 ± 2/29 ± 2			L6; O	[72]
nifedipine	calcium channel blocker; used to treat hypertension and chronic stable angina	100/59.4 *			69.6 ± 16.4/50			MDCKII; O	[71]
spironolactone	antagonist of the mineralocorticoid receptor; treatment of heart failure, hyperaldosteronism, adrenal hyperplasia, hypertension, and nephrotic syndrome	100/36 *			72.2 ± 12.0/50			MDCKII; O	[71]
		50			48 ± 3			MDCKII; O	[73]
tetraethyl-ammonium (TEA)	blocks potassium and voltage-dependent channels; no approved use in humans	448.4 *	211.8 *		50	50		CHO; O	[70]
quinine	target not fully known; antimalarial drug	26.94 *	79.48 *		50	50		CHO; O	[70]
quinidine	blocker of voltage-gated sodium and potassium channels; class I antiarrhythmic agent	17.44 *	45.75 *		50	50		CHO; O	[70]
				500			6.1 ± 0.40	HEK293; O	[60]
				500			≈36.4	Nb2a; E	[24]
lidocaine	blocks sodium channels; local anesthetic, also class Ib antiarrhythmic agent			500			57.1 ± 1.14	HEK293; O	[60]
		100			77.6 ± 7.8			MDCKII; O	[71]
		50			69 ± 7			MDCKII; O	[73]
amiodarone	blocker of voltage gated potassium and voltage gated calcium channels; antiarrhythmic drug	100			66.3 ± 16.2			MDCKII; O	[71]
enalapril	angiotensin-converting enzyme (ACE) inhibitor; treatment of hypertension, heart failure, asymptomatic left ventricular dysfunction and diabetic nephropathy	50			71 ± 4			MDCKII; O	[73]
verapamil	calcium channel blocker; class IV anti-arrhythmia agent used to treat hypertension, angina (chest pain), and certain heart rhythm disorders	17.53 *	46.66 *		50	50		CHO; O	[70]
				500			1.3 ± 0.12	HEK293; O	[60]
				500			≈34	Nb2a; E	[24]
		100/50.9 *			66.5 ± 38.3/50			MDCKII; O	[71]
		50			58 ± 2			MDCKII; O	[73]
		100/1000			54 ± 1/28 ± 1			L6; O	[72]
simvastatine	lipid-lowering drug; treatment of dyslipidemia and to lower the risk of cardiovascular disease	8.457 *	13.05 *		50	50		CHO; O	[70]
pyrilamine	H1 receptor; antihistamine			500			15.4 ± 0.73	HEK293; O	[60]
		500			≈35			SW480; E	[74]
		500			≈27.5			SW480; E	[74]
diphenhydra-mine	H1 receptor; antihistamine, also used for tremor in parkinsonism and as antiemetic			500			43.6 ± 1.74	HEK293; O	[60]
cortisone	naturally occurring glucocorticoid; used in replacement therapy for adrenal insufficiency and as an anti-inflammatory agent	50			64 ± 3			MDCKII; O	[73]
mildronate	inhibitor of gamma-butyrobetaine dioxygenase, inhibits carnitine biosynthesis; anti-ischemia medication	50			42 ± 7			MDCKII; O	[73]
rapamycin/sirolimus	bind the cytosolic protein FK-binding protein 12 (FKBP12) what inhibits the mTOR kinase and blocks activation of T and B cells; immunosuppressive agent indicated for the prevention of transplant rejection	100		100	70.2 ± 5.7		75.2 ± 6.7	HEK293; O	[62]
imatinib	Bcr-Abl tyrosine-kinase inhibitor; cancer treatment (chronic myelogenous leukemia (CML), acute lymphoblastic leukemia (ALL), gastrointestinal stromal tumors (GISTs) and other)	30.99 *	71.19 *		50	50		CHO; O	[70]
vincristine	binds tubulin and stops tubulin dimers from polymerizing to form microtubules; treatment of acute leukaemia, malignant lymphoma, Hodgkin’s disease, acute erythraemia, and acute panmyelosis.	39 *	16.26 *		50	50		CHO; O	[70]
		100		100	33.6 ± 0.1		101 ± 7.3	HEK293; O	[62]
paclitaxel	hyper-stabilizes structure of polymerized microtubules; treatment of ovarian cancer, breast cancer, non-small cell lung carcinoma, Kaposi sarcoma	100		100	64.9 ± 1.8		29.3 ± 8.1	HEK293; O	[62]
daunorubicin	intercalates DNA and inhibits topoisomerase II activity; treatment of acute nonlymphocytic leukemia (myelogenous, monocytic, erythroid)	50		50	62.3 ± 4.5		57.8 ± 2.2	HEK293; O	[62]
vinblastine	binds to the microtubular proteins of the mitotic spindle, preventing polymerisation; treatment of Hodgkin’s disease, lymphocytic lymphoma, advanced testicular cancer, Kaposi’s sarcoma, choriocarcinoma, breast cancer, melanoma	100		100	58.4 ± 1.1		79.9 ± 10.4	HEK293; O	[62]
sunitinib	multi-targeted receptor tyrosine kinase (RTK) inhibitor; treatment of renal cell carcinoma (RCC) and imatinib-resistant gastrointestinal stromal tumor (GIST)	100		100	46.2 ± 1.4		63.6 ± 9.5	HEK293; O	[62]
etoposide	DNA topoisomerase II inhibitor; treatment of testicular cancer, small cell lung cancer, acute myeloid leukaemia, lymphoma, ovarian cancer	100/55 *		100	41.2 ± 4.5/50		64.3 ± 4.1	HEK293; O	[62]
vinorelbine	binds to tubulin and prevents formation of the mitotic spindle; treatment of advanced nonsmall cell lung cancer (NSCLC), metastatic breast cancer	100		100	15.3 ± 1.3		65.6 ± 12.1	HEK293; O	[62]
		60		60	≈20		≈65	HEK293; O	[75]
cisplatin	platinum-based chemotherapy drug that intercalates DNA; treatment of various types of cancers (e.g., small cell lung cancer, metastatic testicular and ovarian cancer, advanced bladder cancer, head and neck epithelial tumors	100		100	≈90		≈65	HEK293; O	[75]
oxaliplatin	cross-links DNA, thus inhibiting DNA synthesis and transcription; treatment of colon and rectum advanced carcinoma	100		100	≈85		≈100	HEK293; O	[75]
cediranib	Vascular endothelial growth factor receptor-2 inhibitor; in development, clinical trials for ovarian cancer, alveolar soft part sarcoma, cervical cancer, endometrial cancer, mesothelioma, prostate cancer and solid tumors	2.49 *			50			HEK293; O	[76]
camptothecin (CZ112)	selectively inhibits the nuclear enzyme DNA topoisomerase, type I; investigated for the treatment of cancer.	10/4.5 ± 1.2			40/50			HEK293; O	[77]
valporate	not fully known; used to treat epilepsy and bipolar disorder and to prevent migraine headaches; has anti-proliferative effects and is the subject of many clinical trials in a variety of cancer types	500			≈60			SW480; E	[74]

* IC50 values for carnitine transport inhibition; O—overexpressed OCTN2/SLC22A5, E—endogenous OCTN2/SLC22A5.

**Table 4:** Analysis of cancer patient survival correlated to SLC22A5 expression.

Cancer Patient Survival Analysis Correlated to *SLC22A5* Expression								
Cancer Type	Prognosis	*p* Value	% 5 Year Survival		n		FPKM Best Cut Off	FPKM Median
			High	Low	High	Low		
glioma	unfavorable	0.0046	6 *	15 *	105	48	1.21	1.51
melanoma	unfavorable	0.16	22 *	53 *	48	54	0.72	0.71
thyroid cancer	unfavorable	0.022	79	95	101	400	1.93	1.59
liver cancer	unfavorable	0.015	42	63	257	108	0.69	0.87
prostate cancer	unfavorable	0.035	97	99	283	211	3.07	3.27
ovarian cancer	unfavorable	0.051	29	35	176	197	1.21	1.18
cervical cancer	unfavorable	0.17	60	73	150	141	1.02	1.04
breast cancer	---	0.39	86	77	600	475	2.54	2.73
pancreatic cancer	favorable	0.00074	61	18	43	133	1.7	1.29
stomach cancer	favorable	0.011	50	27	152	202	1.54	1.45
renal cancer	favorable	0.00084	72	61	599	278	5.25	7.12
endometrial cancer	favorable	2.9 × 10^−5^	81	63	405	136	1.28	1.85
colorectal cancer	favorable	0.022	67	51	411	188	1.49	1.78
head & neck cancer	favorable	0.12	52	41	208	291	0.81	0.75
testis cancer	favorable	0.03	100	94	84	50	0.6	0.74
lung cancer	favorable	0.092	45	45	647	347	0.92	1.06
urothelial cancer	favorable	0.012	47	33	250	156	1.02	1.28

The analysis performed on the data from Protein Atlas (https://www.proteinatlas.org/humanproteome/pathology). FPKM, Fragments per Kilobase of exon per Million reads, * 3-years survival.
